# Pericyte‐derived bone morphogenetic protein 4 underlies white matter damage after chronic hypoperfusion

**DOI:** 10.1111/bpa.12523

**Published:** 2017-05-31

**Authors:** Maiko T. Uemura, Masafumi Ihara, Takakuni Maki, Takayuki Nakagomi, Seiji Kaji, Kengo Uemura, Tomohiro Matsuyama, Raj N. Kalaria, Ayae Kinoshita, Ryosuke Takahashi

**Affiliations:** ^1^ Department of Neurology Kyoto University Graduate School of Medicine Kyoto Japan; ^2^ Department of Neurology National Cerebral and Cardiovascular Center Hospital Osaka Japan; ^3^ Institute for Advanced Medical Sciences Hyogo College of Medicine Hyogo Japan; ^4^ Department of Neurology Ishiki Hospital Kagoshima Japan; ^5^ Institute of Neuroscience Newcastle University, Campus for Ageing and Vitality Newcastle Upon Tyne UK; ^6^ School of Human Health Sciences Kyoto University Graduate School of Medicine Kyoto Japan

## Abstract

Subcortical small vessel disease (SVD) is characterized by white matter damage resulting from arteriolosclerosis and chronic hypoperfusion. Transforming growth factor beta 1 (TGFB1) is dysregulated in the hereditary SVD, CARASIL (cerebral autosomal recessive arteriopathy with subcortical infarcts and leukoencephalopathy). However, very little is known about the role of the largest group in the TGFB superfamily – the bone morphogenetic proteins (BMPs) – in SVD pathogenesis. The aim of this study was to characterize signaling abnormalities of BMPs in sporadic SVD. We examined immunostaining of TGFB1 and BMPs (BMP2/BMP4/BMP6/BMP7/BMP9) in a total of 19 post‐mortem human brain samples as follows: 7 SVD patients (4 males, 76–90 years old); 6 Alzheimer's disease (AD) patients (2 males, 67–93 years old) and 6 age‐matched disease controls (3 males, 68–78 years old). We subsequently investigated the effects of oxygen–glucose deprivation and BMP4 addition on cultured cells. Furthermore, adult mice were subjected to chronic cerebral hypoperfusion using bilateral common carotid artery stenosis, followed by continuous intracerebroventricular infusion of the BMP antagonist, noggin. In the SVD cases, BMP4 was highly expressed in white matter pericytes. Oxygen–glucose deprivation induced BMP4 expression in cultured pericytes *in vitro*. Recombinant BMP4 increased the number of cultured endothelial cells and pericytes and converted oligodendrocyte precursor cells into astrocytes. Chronic cerebral hypoperfusion *in vivo* also upregulated BMP4 with concomitant white matter astrogliogenesis and reduced oligodendrocyte lineage cells, both of which were suppressed by intracerebroventricular noggin infusion. Our findings suggest ischemic white matter damage evolves in parallel with BMP4 upregulation in pericytes. BMP4 promotes angiogenesis, but induces astrogliogenesis at the expense of oligodendrocyte precursor cell proliferation and maturation, thereby aggravating white matter damage. This may explain white matter vulnerability to chronic hypoperfusion. The regulation of BMP4 signaling is a potential therapeutic strategy for treating SVD.

## INTRODUCTION

Vascular cognitive impairment develops as a consequence of various types of cerebrovascular alterations. Subcortical white matter changes caused by small vessel alterations are frequently observed in vascular cognitive impairment and are referred to as subcortical ‘small vessel disease (SVD)’ 39. Disturbances in cerebrospinal fluid production 40, cerebral edema 22, breakdown of the blood–brain barrier and increased permeability 21, 54, oxidative stress 4, and inflammation have been cited as important causes in the development of white matter changes 19. However, the exact mechanisms have yet to be fully elucidated. Recent studies have noted that attenuations of vasculature and white matter are also frequently observed in other neurodegenerative disorders, especially in Alzheimer's disease (AD) 46, 59. However, vascular risk factors are related to a lesser degree to a pure type of AD 10, 38 or mixed type (AD with vascular pathology) dementia 23 compared with vascular cognitive impairment. The involvement of different etiologies has been suggested in white matter damage between SVD and AD 13, 20. Therefore, clarification of causative factors that underlie various forms of white matter changes should enable the development of novel strategies for tackling cognitive impairment.

There are several recognized forms of inherited SVD 9, including CARASIL (cerebral autosomal recessive hereditary cerebral artery disease and arteriosclerosis with subcortical infarcts and leukoencephalopathy). The gene responsible for CARASIL is *HTRA1* (high‐temperature requirement A 1), and the resultant upregulation of transforming growth factor beta (TGFB) family signaling is postulated to underlie the small vessel changes observed in CARASIL 18. The TGFB superfamily also includes the bone morphogenetic protein (BMP) family proteins.

BMPs are involved in oligovascular pathologies in the ischemic brain, in addition to their physiological roles in embryonic and bone tissues. In an ischemic intrauterine growth retardation model, oxidative stress upregulates BMP4 and mediates periventricular white matter injury with a paucity of mature oligodendrocytes and hypomyelination, while BMP deletion reverses these defects 36. In neonatal mouse brains, BMP4 expression increases as a consequence of global hypoxia‐ischemia, while the BMP antagonist noggin protects the white matter against damage 12. Furthermore, in adult mouse brains with focal cerebral ischemia, overexpressed noggin reduces infarct volume and motor deficits 44. These experimental results highlight the potential of BMP antagonism in the treatment of ischemic demyelinating disorders.

Nevertheless, the exact role of BMP family members remains poorly understood in the context of chronic hypoperfusion in the adult human brain. Therefore, the present study explored the underlying etiology of white matter abnormalities, specifically focusing on BMP expression through the use of postmortem human brains, cultured cells exposed to oxygen–glucose deprivation (OGD), and chronic hypoperfusion mouse brains.

## MATERIALS AND METHODS

### Experimental design of postmortem brain material

Study samples were selected from 371 autopsied brains retained at Kyoto University Hospital from 1983 to 2009. This collection was approved by the institutional research committee, Kyoto University Graduate School and Faculty of Medicine, Ethics Committee. Neuropathological diagnoses were made according to thorough histopathological examination of extensively sampled brain sections, as previously described 1, 35. Cases with mixed SVD and AD pathological diagnoses were excluded to select for brain samples with relatively single processes. We analyzed a total of 19 postmortem brain samples as follows: 7 SVD patients (4 males, 76–90 years old, brain weight 1101 ± 89.9 g); 6 AD patients (2 males, 67–93 years old, brain weight 1056 ± 67.7 g) and 6 age‐matched disease controls (3 males, 68–78 years old, brain weight 1190 ± 113.7 g). Table 1 provides demographics and pathological features of the 19 subjects. The clinical diagnosis of dementia met the criteria of the Diagnostic and Statistical Manual of Mental Disorders IV 2. The AD neuropathological diagnoses were made according to the presence of frequent senile plaques in the neocortex 32, and no less than stage V of Braak stage of neurofibrillary tangles 6, 7. The diagnosis of SVD, or subcortical ischemic vascular dementia was clinically made 5 and was retrospectively found to meet the pathological criteria outlined by Kalaria *et al* 24. Two observers (M.T.U. and M.I.) individually assessed senile plaque and neurofibrillary tangle stages, and if required, a joint assessment was scrutinized under a two‐headed microscope.

**Table 1:** Demographics of subjects and pathological analysis.

	No.	Age	Sex	BW(g)	SP	NFT	FMI	Clinical and pathological diagnosis
Control	1	68	M	1400	None	0	79.7	ALS
	2	71	F	1280	Sparse	I	78.0	ALS
	3	75	M	1090	Sparse	I	97.1	Emphysema
	4	70	F	1030	None	0	72.4	Schizophrenia
	5	80	F	1150	Sparse	I	67.8	ALS
	6	79	M	–	Sparse	I	90.2	ALS
AD	7	80	F	–	Frequent	V	54.0	AD
	8	85	M	1060	Frequent	IV	79.5	AD
	9	67	F	940	Frequent	VI	74.4	AD
	10	84	F	1080	Frequent	VI	67.6	AD
	11	93	F	1150	Frequent	VI	74.8	AD
	12	77	M	1050	Frequent	VI	78.5	AD
SVD	13	80	M	1120	None	0	46.5	SVD
	14	70	M	1200	Sparse	II	61.6	SVD
	15	86	F	940	Sparse	III	55.9	SVD
	16	76	F	1100	None	III	59.9	SVD
	17	90	M	–	Sparse	III	47.8	SVD
	18	82	M	1050	Sparse	I	53.5	SVD
	19	82	F	1200	Sparse	III	68.5	SVD

AD = Alzheimer's disease; ALS = amyotrophic lateral sclerosis; BW = brain weight; FMI = frontal myelin index; NFT = neurofibrillary tangle; SP = senile plaque; SVD = small vessel disease.

### Tinctorial staining and immunohistochemistry for postmortem human brain samples

Six micrometer‐thick paraffin‐embedded tissue sections were cut from the frontal lobes at the level of the olfactory bulbs and stained for Luxol fast blue. Some sections were also incubated with specific primary antibodies (listed in *Supporting Information* Table S1) overnight at 4°C. Sections were pretreated at 90°C for 20 min in Tris‐ethylenediamine tetraacetic acid (pH 9.0) for actin, alpha 2, smooth muscle, aorta (ACTA2, also known as αSMA) and platelet‐derived growth factor receptor alpha (PDGFRA), and some were pretreated at 121°C for 15 min in 0.01 M citrate buffer (pH 6.0) using an autoclave for BMP2. This was followed by appropriate biotinylated secondary antibody (1:200, Vector Laboratories, Burlingame, CA, USA) and avidin‐biotin‐peroxidase complex (1:200, Vector Laboratories), or polymer Detection System (Histofine Simple Stain MAX PO MULTI, Nichirei Biosciences, Tokyo, Japan) application. Between steps, the sections were washed three times for 5 min with 0.1 M phosphate‐buffered saline (PBS, pH 7.4) and visualized using 0.01% diaminobenzidine tetrahydrochloride and 0.003% H_2_O_2_ in 50 mM Tris‐HCl (pH 7.5).

For triple‐immunofluorescence, deparaffinized sections were incubated with a mixture of primary antibodies overnight at 4°C, followed by Alexa Fluor 488, 594 and 647 conjugated secondary antibodies (1:200, Invitrogen, Carlsbad, CA, USA) for 2 h at room temperature.

The images of interest were captured with a microscope (BZ‐X700, Keyence, Osaka, Japan) or a confocal laser‐scanning microscope (FV1000, Olympus, Tokyo, Japan).

### Assessment of myelin density or immunohistochemical staining

**Figure 1:**
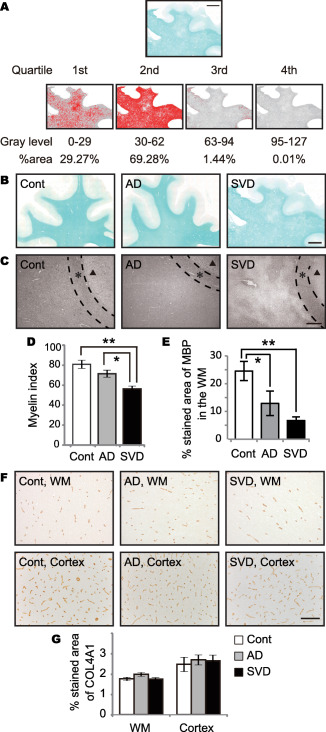
Degree of myelin loss and capillary bed density in the frontal lobes of post‐mortem human brain samples. **A.** A procedure for calculating myelin index. The median gray level of each quartile (14.5, 46.0, 78.5 and 111.0) is multiplied by percent area in each quartile (29.27%, 69.28%, 1.44% and 0.01%), and summed up to be 37.25 as the myelin index for the representative image of LFB staining. *Bar* indicates 5 mm. **B.** Representative images of LFB staining in control, AD, and SVD cases. *Bar* indicates 5 mm. **C.** Representative images of MBP staining (myelin) in control, AD, and SVD cases. *Bar* indicates 400 µm. *Black triangles* indicate cortex. *Asterisks* indicate U‐fiber. **D.** Myelin index calculated from each LFB staining of all three groups. Differences are significant between any two groups, except between control and AD: control vs. AD, *P* = 0.226; control vs. SVD, **P = 0.001; AD vs. SVD, *P = 0.027. **E.** The percentage of MBP in the WM in all three groups. Differences are significant between any two groups, except between AD and SVD: control vs. AD, *P = 0.027; control vs. SVD, **P = 0.001; AD vs. SVD, *P* = 0.224. **F.** Representative images of COL4A1 staining (basement membrane marker) in control, AD, and SVD cases. *Bar* indicates 100 µm. **G.** The percentage of COL4A1 in the WM and cortex in all three groups. Differences are not significant in the WM (*P* = 0.095) and cortex (*P* = 0.095). *Vertical bars* represent mean ± SEM. Abbreviations are as follows: AD, Alzheimer's disease; Cont, control; MBP, myelin basic protein; LFB, Luxol fast blue; SVD, small vessel disease; WM, white matter.

Myelin density was assessed using immunohistochemistry and myelin index as previously described 20, 58. Briefly, to determine the myelin index, the white matter was automatically outlined using the wand tool of Image J. If the border between the cortex and white matter was obscured, the white matter was manually outlined using the semiautomatic trace tool instead of the wand tool. The detected range of gray levels within the white matter, corresponding to the staining intensity, from 0 to 127 (0, white; 255, black) was divided into four quartiles (the first quartile 0–29, the second 30–62, the third 63–94 and the fourth 95–127). The percent area for each quartile was calculated. The median gray level of each quartile (14.5, 46.0, 78.5 and 111.0), an estimated staining intensity, was then multiplied by percent area in each quartile and summed up, providing the total myelin index (Figure 1A).

For immunohistochemical staining, images from 15 to 20 randomly selected ROIs were captured. The images were then converted into 8‐bit grayscale, binarized by threshold using the Triangle Method, and the area above threshold measured using Fiji and Image J. For quantification of BMP4 immunoreactivity in PDGFRB‐ and ACTA2‐positive cells, images of PDGFRB or ACTA2 immunostaining were firstly converted into binarized images using the Triangle Method and the area above threshold was selected. The selected area was applied to the same section with BMP4 immunostaining, and the mean intensity and percent area of BMP4 in the selected area measured.

The above analyses were performed blinded to the diagnosis by labeling sections with arbitrary numbers.

### Cell culture

Mouse brain vascular pericytes (1200, ScienCell, Carlsbad, CA, USA) and human brain microvascular endothelial cells (ACBRI, HBMDC, Cell Systems, Kirkland, WA, USA) were cultured with pericyte medium (1201, ScienCell) or endothelial cell growth medium (CC‐3156 and CC‐4147, Lonza, Basel, Switzerland) containing 10% fetal bovine serum and 1% penicillin/streptomycin. Endothelial cells were placed on pre‐coated dishes with 3.2% bovine collagen type IV alpha 1 chain (COL4A1) (A1064401, Invitrogen) in PBS. Oligodendrocyte precursor cells (OPCs) were isolated from cerebral cortices from 1‐ to 2‐day‐old Sprague Dawley rats as previously described 8, 30.

### Oxygen–glucose deprivation

Upon confluency, pericyte media were replaced with glucose‐ and serum‐free pericyte media. The following day, culture plates were placed into a hypoxic culture kit (BIONIX II, Sugiyama‐Gen, Tokyo, Japan) with 5% O_2_ and 3% CO_2_, and incubated at 37°C for 1, 2 and 3 days.

### RT‐PCR

After preparing the cell lysate, mRNA was rapidly purified using the RNeasy Plus Mini Kit (74136, Qiagen, Hilden, Germany) and subjected to reverse transcriptase‐polymerase chain reaction (RT‐PCR) analysis using appropriate primers (listed in *Supporting Information* Table S2).

### BMP4 and noggin treatment

The media were replaced with new medium with or without recombinant human BMP4 (1 or 10 ng/ml, 314‐BP, R&D systems) and/or recombinant human noggin (100 ng/ml for proliferation assay and 500 ng/ml for differentiation assay; 120–10C, PeproTech, IL, USA). The cells treated with both BMP4 and noggin were pretreated with only noggin (100 ng/ml for proliferation assay and 500 ng/ml for differentiation assay) for 2 h.

### WST‐8 assay

Two days after BMP4 and/or noggin administration, 1% of 2‐(2‐methoxy‐4‐nitrophenyl)‐3‐(4‐nitrophenyl)‐5‐(2,4‐disulfophenyl)‐2H‐tetrazolium, monosodium salt solution (WST‐8, 07553–44, Nacalai Tesque, Kyoto, Japan) was added to each medium. Then, the plates were incubated for 1 h at 37°C in the incubator. Absorbance at 450 nm and 630 nm were measured using a multi‐label plate reader (2030 ARVO X, PerkinElmer, Waltham, MA, USA). The results of absorbance at 450 nm were corrected to absorbance at 630 nm.

### Immunocytochemistry

The cells were fixed with 4% paraformaldehyde/PBS for 15 min at room temperature, treated with 3% bovine serum albumin for 1 h, incubated with primary antibodies (listed in *Supporting Information* Table S3) overnight at 4°C and appropriate secondary antibodies for 2 h at room temperature. Between steps, the cells were washed three times for 5 min with PBS.

### Tube formation assay

After thawing overnight at 4°C, the matrigel (354230, BD Biosciences, Franklin Lakes, NJ, USA) was applied on each well of a μ‐slide (81506, Ibidi, Madison, WI, USA) and polymerized for 1 h at 37°C. Then, 50 μl of suspension at a density of 4 × 10^5^ cells/ml endothelial cells were added to the matrigel surface. After seeding the cells, the slides were incubated for 24 h at 37°C. The number of tubes was quantified under a microscope.

### OPC proliferation assay and differentiation assay

For the proliferation assay, OPCs were cultured with OPC proliferation media (Neurobasal medium containing 2 mM glutamine, 1% penicillin/streptomycin, 10 ng/ml platelet‐derived growth factor‐AA, 10 ng/ml basic fibroblast growth factors and 2% B27 supplement). At 30% confluency, the cells were treated with BMP4 and/or noggin and incubated for 2 days until harvest and analysis by RT‐PCR.

For the differentiation assay, when the cells were 70% confluent, the OPC proliferation media were replaced with OPC differentiation media (Dulbecco's‐modified Eagle Medium containing 1% penicillin/streptomycin, 10 ng/ml ciliary neurotrophic factor, 10 ng/ml triiodothyronine and 2% B27 supplement). Simultaneously, BMP4 and/or noggin were administered. Four days after treatment, the cells were analyzed by immunocytochemistry or RT‐PCR.

Changes in OPC morphologies were captured using a time‐lapse camera (BZ‐X700, Keyence).

### Animals and surgical procedure

The protocol for this study was approved by the Institutional Animal Care and Use Committee, Institute of Laboratory Animals Graduate School of Medicine, Kyoto University.

Adult C57BL/6J male mice (10–12 weeks old) were subjected to bilateral common carotid artery stenosis (BCAS) using microcoils as previously described (n = 6) 29, 48. Control mice underwent sham surgery (n = 6). Four weeks after BCAS, mice were euthanized and their brains analyzed by immunohistochemistry and western blot (Figure 6A).

In a separate experiment, mice received continuous intracerebroventricular infusion (cICV) 2 days prior to BCAS using brain infusion kit 3 (0008851, Alzet, Cupertino, CA, USA) and micro‐osmotic pump (1004, Alzet) under stereotaxis. The pumps were filled with recombinant human noggin (500 ng/day or 1000 ng/day) in artificial cerebrospinal fluid (ACSF, 3525, Funakoshi, Tokyo, Japan), or ACSF only, as previously described 42, 43. Control mice underwent sham surgery (n = 6 for sham control; n = 6 for BCAS and ACSF; n = 5 for BCAS and noggin 500 ng/day; n = 6 for BCAS and noggin 1000 ng/day) (Figure 6H).

### Immunohistochemistry for mouse brain

Mice were anesthetized and intracardially perfused with cooled PBS. The brains were quickly frozen using powdered dry ice. Coronal sections (20‐μm thick) were cut on a cryostat at −20°C and collected onto glass slides. As previously described 47, sections were fixed with 4% paraformaldehyde/PBS for 15 min and incubated with antibodies (listed in *Supporting Information* Table S4). Quantitative analyses of glial fibrillary acidic protein (Gfap)‐positive astrocytes, oligodendrocyte transcription factor (Olig2)‐positive oligodendrocyte lineage cells, and myelin basic protein (Mbp)‐positive mature oligodendrocytes were performed in the bilateral paramedian corpus callosum using Image J.

### Western blot

Western blot analysis was performed as previously described 28. Briefly, the dorsal half of the brain at 0–0.5 mm anterior to bregma was homogenized in radio‐immunoprecipitation assay buffer, followed by sonication for 5 min with a 30‐s interval and centrifugation at 14 000 rpm for 5 min at 4°C. The supernatant was boiled in sample buffer (1% (w/v) sodium dodecyl sulfate (SDS), 12.5% (w/v) glycerol, 0.005% (w/v) bromophenol blue and 2.5% (v/v) 2‐mercaptoethanol in 25 mM Tris‐HCl, pH 6.8). Samples were separated onto 10% (w/v) gels for SDS‐PAGE or 4%–12% (w/v) gels for NuPAGE (NP032330X, Invitrogen), followed by transfer to polyvinylidene difluoride membranes (Millipore, Billerica, MA, USA). The membranes were then incubated overnight at 4°C with primary antibodies (listed in *Supporting Information* Table S5) followed by the appropriate horseradish peroxidase‐conjugated secondary antibodies for 1 h at room temperature. Immunoreactive bands were detected with chemiluminescence assay kits (02230, Nacalai Tesque), ECL Western Blotting Substrate (32106, Thermo Fisher Scientific, Waltham, MA, USA), and Amersham Imager 600 (GE Healthcare, Buckinghamshire, UK).

### Statistical analysis

Statistical analysis was performed using the R statistical package (www.r-project.org). Statistical significance was evaluated using two‐tailed paired Student's *t*‐test, the Wilcoxon rank‐sum test or one‐way ANOVA followed by the Bonferroni post‐hoc test. Pearson correlation analysis was performed to observe a possible correlation. For all analyses, the level of statistical significance was set at **P < 0.01 or *P < 0.05.

## RESULTS

### Myelin loss in SVD and AD compared with controls

Luxol fast blue staining (Figure 1B) and MBP immunohistochemical staining (Figure 1C) represented severe myelin attenuation in the SVD group. The myelin index was significantly different between the SVD group and the other two groups as follows: controls (80.9) > AD (71.5) > SVD (56.3) (Figure 1D), indicating that myelin loss was greatest in the SVD group. Age did not correlate with myelin index (*r* = −0.329, *P* = 0.170, data not shown). The percent area of MBP immunoreactivity also decreased in the SVD group (Figure 1E).

### Capillary bed density

Capillary bed density was determined by immunohistochemistry using antibody specific to COL4A1, a major constituent of the basement membrane (Figure 1F). Quantitative analysis showed that the percentage of COL4A1 density was not different in the white matter or cortex between the three groups (white matter, *P* = 0.095; cortex, *P* = 0.095) (Figure 1G).

### Pericyte density in the postmortem human brain

Pericyte density was assessed by two different markers: ACTA2 and platelet‐derived growth factor receptor beta (PDGFRB). The percentage of ACTA2 and PDGFRB expression, respectively, were divided by the percentage of COL4A1 stained area to correct for vascular density.

**Figure 2:**
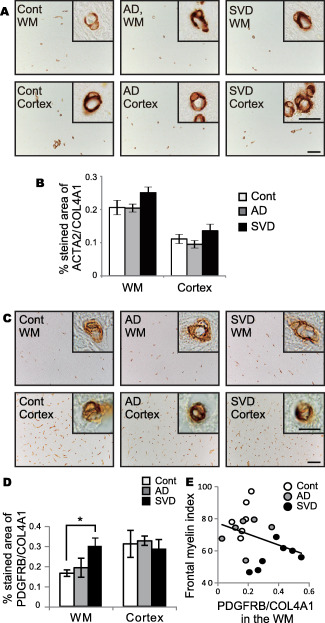
Pericyte density as assessed by ACTA2 and PDGFRB labeling in the post‐mortem human brain samples. **A.** Representative images of ACTA2 staining in control, AD, and SVD cases. Insets show ACTA2‐positive pericytes. *Bars* indicate 100 µm and 10 µm (insets). **B.** The percentage of ACTA2 in the WM and cortex in all three groups. Differences are not significant in the WM (*P* = 0.123) and cortex (*P* = 0.220). **C.** Representative images of PDGFRB staining in control, AD, and SVD cases. Insets show PDGFRB‐positive pericytes. *Bars* indicate 100 µm and 10 µm (insets). **D.** The percentage of PDGFRB in the WM and cortex in all three groups. Differences are significant between control and SVD in the WM: control vs. AD, *P* = 0.955; control vs. SVD, *P = 0.046; AD vs. SVD, *P* = 0.081. Differences are not significant in the cortex (*P* = 0.464). **E.** An inverse correlation between the percentage of PDGFRB and the myelin index all 19 cases combined (*r* = −0.568, *P = 0.013). *Vertical bars* represent mean ± SEM. Abbreviations are as follows: AD, Alzheimer's disease; Cont, control; SVD, small vessel disease; WM, white matter.

The ACTA2 immunoreactivity was not different in the white matter and cortex between the three groups (white matter, *P* = 0.123; cortex, *P* = 0.220) (Figure 2A,B). Conversely, the percentage of PDGFRB expression was significantly increased in white matter of the SVD group compared with the other two groups (Figure 2C,D). There was a significant inverse correlation between the percentage of PDGFRB/COL4A1 expression in the white matter and myelin index in all 19 cases combined (*r* = −0.568, *P = 0.013) (Figure 2E).

### BMP4 expression in PDGFRB‐positive pericytes of damaged white matter in the postmortem human brain

**Figure 3:**
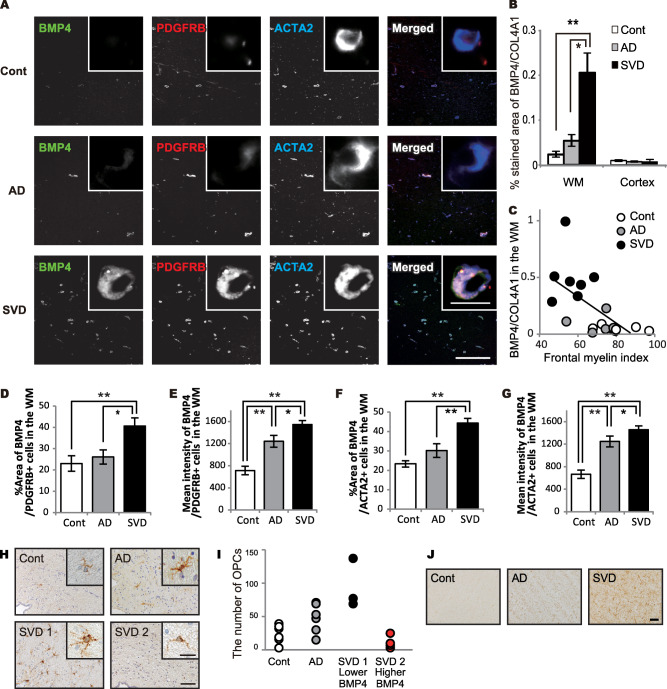
Pericyte BMP4 expression in damaged white matter in the post‐mortem human brain samples. **A.** Representative triple‐immunofluorescent images for BMP4, PDGFRB, and ACTA2, and their merged images in the WM of all three groups. Insets show enlarged pericytes. *Bars* indicate 400 µm and 10 µm (insets). **B.** The percentage of BMP4 expression in all three groups. Differences are significant between any two groups, except between control and AD in the WM: control vs. AD, *P* = 0.804; control vs. SVD, **P = 0.003; AD vs. SVD, *P = 0.012. Differences are not significant in the cortex (*P* = 0.659). **C.** An inverse correlation between the percentage of BMP4 expression and the myelin index in all 19 cases combined (*r* = −0.549, *P = 0.015). **D.** The percent area of BMP4 expression in PDGFRB‐positive cells in the WM of all three groups. Differences are significant between any two groups, except between control and AD: control vs. AD, *P* = 0.593; control vs. SVD, **P = 0.001; AD vs. SVD, *P = 0.011. **E.** The mean intensity of BMP4 in PDGFRB‐positive cells in the WM of all three groups. Differences are significant between any two groups: control vs. AD, **P < 0.001; control vs. SVD, **P < 0.001; AD vs. SVD, *P = 0.017. **F.** The percent area of BMP4 expression in ACTA2‐positive cells in the WM of all three groups. Differences are significant between any two groups, except between control and AD: control vs. AD, *P* = 0.079; control vs. SVD, **P < 0.001; AD vs. SVD, **P < 0.001. **G.** The mean intensity of BMP4 in ACTA2‐positive cells in the WM of all three groups. Differences are significant between any two groups: control vs. AD, **P < 0.001; control vs. SVD, **P < 0.001; AD vs. SVD, *P = 0.018. **H.** Representative images of PDGFRA expression in the interstitium of the SVZ in the cases of control, AD, and SVD of post‐mortem human brain samples. According to expression levels of BMP4 (BMP4‐to‐COL4A1 ratio), the SVD cases are further classified into subgroup: a lower BMP‐4 expressing group (SVD 1) and a higher one (SVD 2) with the cut‐off ratio of 0.31. Insets show enlarged images of OPCs. *Bars* indicate 50 µm and 10 µm (insets). **I.** The number of OPCs in all 4 groups. SVD 2 group shows smaller number of OPCs compared with SVD 1 group. **J.** Representative images of GFAP expression in the WM of post‐mortem human brain samples. The WM of SVD shows severe astrogliosis with high GFAP expression. *Bar* indicates 50 µm. *Vertical bars* represent mean ± SEM. Abbreviations are as follows: AD, Alzheimer's disease; Cont, control; SVD, small vessel disease; SVZ, subventricular zone; WM, white matter.

BMP4 was strongly expressed in abluminal surface of capillaries and tunica media of arterioles, and mildly expressed in macrophages, and activated astrocytes in the white matter of SVD brains. Triple immunofluorescence studies revealed abundant BMP4 expression in PDGFRB‐ and ACTA2‐positive pericytes in the white matter of SVD cases. However, in control or AD brains, BMP4 expression was weak (Figure 3A).

Similar to the percentage of PDGFRB/COL4A1 expression, the percentage of BMP4/COL4A1 expression was significantly increased in the white matter of SVD cases but was not different in the cortex between the three groups (Figure 3B). BMP4 expression negatively correlated with the myelin index (*r* = −0.549, *P = 0.015) (Figure 3C) in all 19 cases combined. Fluorescence intensity and percentage of BMP4‐positive area in PDGFRB‐ and ACTA2‐positive cells were increased in SVD cases (Figure 3D–G), suggesting BMP4 expression was increased in pericytes. BMP4 expression was also observed in activated astrocytes and macrophages in SVD, but not control or AD, cases (data not shown).

### Other TGFB superfamily members (TGFB1, BMP2, BMP6, BMP7 and BMP9)

Macrophages and activated astrocytes within the vicinity of microinfarcts were immunoreactive to all six TGF superfamily members. BMP2 was highly expressed in the endothelial cells of arterioles, venules and capillaries, and pericytes of capillaries in all three groups. BMP7 and TGFB1 were slightly expressed in the tunica media of arterioles and capillaries. BMP6 and BMP9 were not expressed in blood vessels (*Supporting Information* Figure S1).

### Oligodendrocyte precursor cells and astrocytes in the postmortem human brain

PDGFRA‐positive OPCs were found in the subventricular zone (SVZ), and cellular processes of some OPCs were found around arterioles, venules and capillaries (Figure 3H, *Supporting Information* Figure S2). The number of brain interstitial OPCs was measured in the SVZ of all cases and found to be increased relative to white matter attenuation, as previously described 33. However, when the SVD cases were divided into two groups by the cut‐off point of 0.31 for BMP4/COL4A1, the higher BMP4/COL4A1 group (SVD 2, n = 4) tended to have a smaller number of OPCs than the lower BMP4/COL4A1 group (SVD 1, n = 3) (Figure 3I). The GFAP‐positive astrocytes were increased in the white matter of SVD cases (Figure 3J).

### BMP4 expression in cultured pericytes under oxygen–glucose deprivation

**Figure 4:**
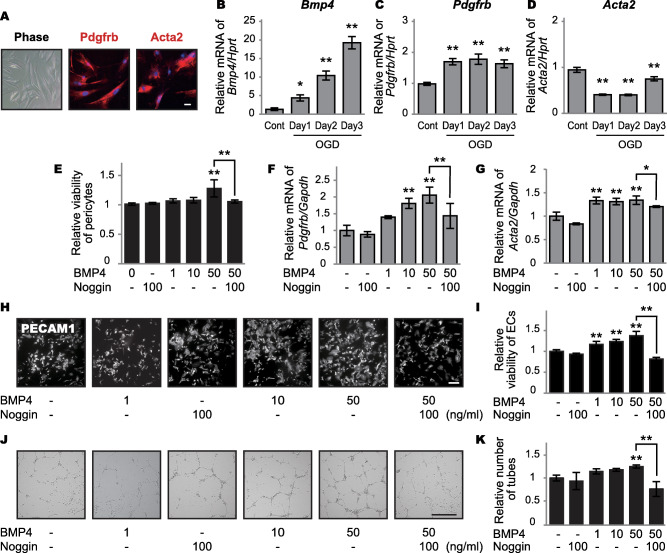
Effects of BMP4 expression on cultured endothelial cells and pericytes. **A.** Representative images of mouse pericyte cell line. The cells express Pdgfrb and Acta2, both established pericyte markers. *Bar* indicates 50 µm. **B–D.** Relative mRNA levels of *Bmp4* (B), *Pdgfrb* (C) and *Acta2* (D) in cultured pericytes under OGD. *Bmp4* mRNA levels show a time‐dependent increase with continuous OGD for 3 days (*P = 0.024 for Day 1 and **P < 0.001 for Days 2 and 3). *Pdgfrb* mRNA levels are increased (**P < 0.001 for Days 1, 2 and 3), but *Acta2* mRNA levels are decreased (**P < 0.001 for Days 1, 2 and 3) by OGD. The expression of each mRNA sample is normalized to *Hprt* expression, which does not fluctuate under continuous OGD. **E.** Relative viability of pericytes as assessed by WST‐8 assay. High‐dose BMP4 increases pericyte viability (**P < 0.001 for 50 ng/ml), which is blocked by noggin (**P = 0.009). **F, G.** Relative mRNA levels of *Pdgfrb* and *Acta2* in cultured pericytes after BMP4 administration. BMP4 increases *Pdgfrb* mRNA levels in a dose‐dependent manner (**P = 0.001 for 10 ng/ml and **P < 0.001 for 50 ng/ml), which is blocked by noggin (**P = 0.009) (F). BMP4 increases *Acta2* mRNA levels (**P < 0.001 for 1, 10 and 50 ng/ml), which is blocked by noggin (**P = 0.002) (G). **H**. Representative images of PECAM1‐positive endothelial cells after additions of BMP4 with or without noggin. *Bar* indicates 50 µm. **I**. Relative viability of endothelial cells as assessed by WST‐8 assay. BMP4 increases viability of endothelial cells in a dose‐dependent manner (**P < 0.001 for 1, 10 and 50 ng/ml), which is blocked by noggin (**P < 0.001). **J.** Representative images of endothelial cell tube formation after BMP4 additions with or without noggin. *Bar* indicates 500 μm. **K.** BMP4 facilitates tube formation of endothelial cells (**P = 0.008 for 50 ng/ml), which is blocked by noggin (**P < 0.001). *Vertical bars* represent mean ± SD. Abbreviations are as follows: ECs, endothelial cells; Gapdh, glyceraldehyde‐3‐phosphate dehydrogenase; Hprt, hypoxanthine‐guanine phosphoribosyltransferase expression; OGD, oxygen–glucose deprivation; PECAM1, platelet and endothelial cell adhesion molecule 1, also known as CD31.

To confirm whether BMP4 is increased in pericytes under chronic hypoperfusion, we analyzed *Bmp4* mRNA expression in cultured pericytes under continuous OGD. The mouse brain microvascular pericyte cell line expressed both Pdgfrb and Acta2 (Figure 4A). *Bmp4* mRNA expression exhibited a time‐dependent increase under continuous OGD for 3 days (Figure 4B). *Pdgfrb*, but not *Acta2*, mRNA expression increased under OGD (Figure 4C,D). Expression of each mRNA sample was normalized to hypoxanthine‐guanine phosphoribosyltransferase expression (*Hprt*) expression, which did not fluctuate under continuous OGD.

### Effects of BMP4 on pericyte proliferation and *Pdgfrb/Acta2* mRNA expression

The WST‐8 assay showed that high‐dose BMP4 induced pericyte proliferation (Figure 4E). BMP4 also increased *Pdgfrb* (Figure 4F) and *Acta2* (Figure 4G) mRNA expression levels.

### Effects of BMP4 on endothelial cell proliferation and tube formation

Endothelial cell proliferation in response to BMP4 treatment was assessed by immunocytochemistry (Figure 4H) and WST‐8 assay (Figure 4I). Endothelial cell proliferation and tube formation (Figure 4J,K) were facilitated in a dose‐dependent manner by BMP4, but were blocked by the BMP4 antagonist, noggin.

### Effects of BMP4 on oligodendrocyte precursor cells

**Figure 5:**
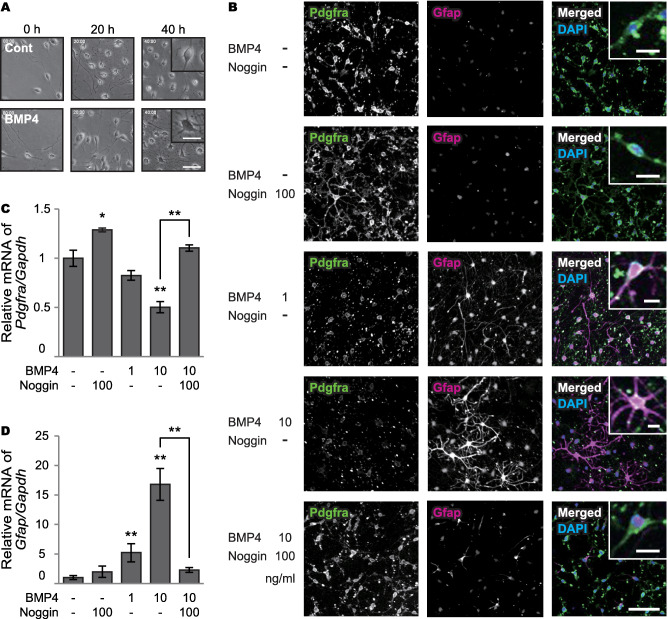
Effects of BMP4 on cultured oligodendrocyte precursor cells. **A.** Time‐lapse images of primary OPCs in proliferation media with or without BMP4. Insets show enlarged images. *Bars* indicate 100 μm and 50 μm (insets). **B.** Representative triple‐immunofluorescent images for Pdgfra and Gfap, and their merged images with DAPI for nuclear staining. Insets show enlarged images. *Bars* indicate 50 µm and µm (insets). **C, D.** Relative mRNA levels of *Pdgfra* and *Gfap* in the proliferation assay. (C) BMP4 decreases *Pdgfra* mRNA levels in a dose‐dependent manner (*P* = 0.062 for 1 ng/ml and **P < 0.001 for 10 ng/ml), which is blocked by noggin (**P = 0.001). Treatment with noggin alone increases *Pdgfra* mRNA levels (*P = 0.015). (D) BMP4 significantly increases *Gfap* mRNA expression in a dose‐dependent manner (**P = 0.004 for 1 ng/ml and **P < 0.001 for 10 ng/ml), which is blocked by noggin (**P < 0.001). *Vertical bars* represent mean ± SD. Abbreviations are as follows: Cont = control; Gapdh = glyceraldehyde‐3‐phosphate dehydrogenase.

In the OPC proliferation assay, treatment of BMP4 converted OPCs into astrocyte‐like cells, with thick and long processes (Figure 5A and *Supporting Information* Movie S1). Immunocytochemistry showed that BMP4 treatment decreased Pdgfra (OPC marker) immunoreactivities and increased Gfap (astrocyte maker) (Figure 5B). RT‐PCR also showed that BMP4 decreased *Pdgfra* mRNA levels, and significantly increased *Gfap* mRNA levels in a dose‐dependent manner; noggin blocked the conversion (Figure 5C,D). Treatment with noggin alone increased *Pdgfra* mRNA levels in OPCs due to inhibition of endogenous BMP4 (Figure 5C).

The effects of BMP4 on OPCs were also observed in the OPC differentiation assay. In the differentiation media, primary OPCs differentiated into mature oligodendrocytes, whereas BMP4 treatment induced astrocyte‐like cells (*Supporting Information* Figure S3A and Movie S2). BMP4 treatment decreased Mbp (oligodendrocyte marker) and increased Gfap in a dose‐dependent manner; noggin also blocked the conversion (*Supporting Information* Figure S3B–D).

### Bmp4 expression and effect of noggin in the mouse brain under chronic hypoperfusion

**Figure 6:**
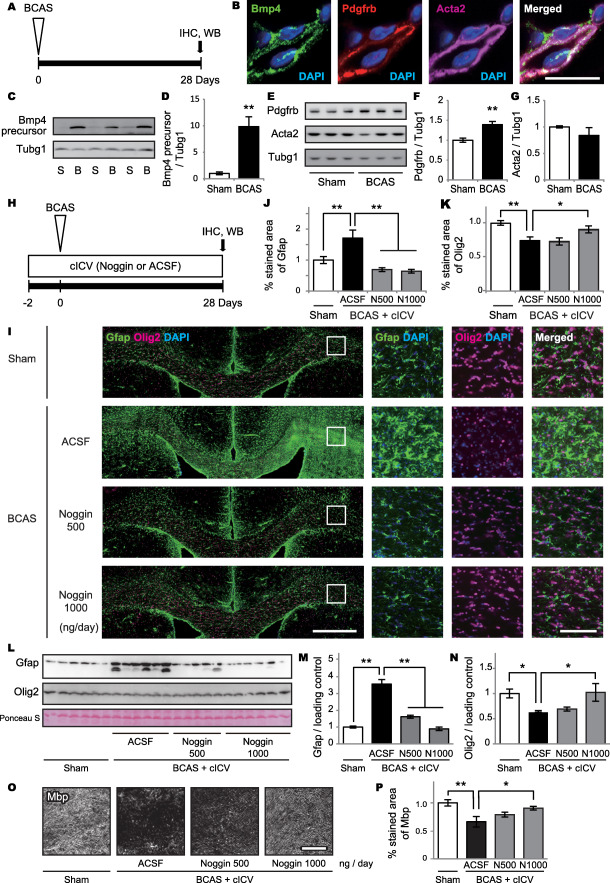
BMP4 expression and effect of noggin in the mouse brain after chronic hypoperfusion. **A.** Experimental design of the first trial. Adult mice subjected to BCAS are analyzed by immunohistochemistry and western blot. **B.** Representative triple‐immunofluorescent images for Bmp4, Pdgfrb and Acta2, and their merged images with DAPI for nuclear staining. *Bar* indicates 10 µm. **C–G.** Western blot shows significantly increased Bmp4 precursor (C, D) and Pdgfrb (E, F) levels after BCAS (**P = 0.002 for Bmp4 precursor and **P = 0.004 for Pdgfrb), without significant changes in Acta2 levels (*P* = 0.660) (E, G). **H.** Experimental design of the second trial. Adult mice subjected to BCAS receive noggin (500 ng/day or 1000 ng/day) in ACSF or ACSF only through cICV 2 days prior to BCAS, are euthanized and their brains analyzed by immunohistochemistry and western blot. **I.** Representative immunofluorescent images of Gfap and Olig2 with DAPI (the leftmost images) and their enlarged images (three right images). *Bars* indicate 500 µm (left) and 100 µm (right). **J.** Semiquantitative analysis of immunofluorescent images shows that BCAS significantly increases GFAP‐positive cells (**P = 0.004), which is strongly suppressed by noggin cICV (**P < 0.001 for both 500 ng/day and 1000 ng/day). **K.** Semiquantitative analysis of immunofluorescent images shows BCAS significantly decreases Olig2‐positive cells (**P < 0.001), which is significantly ameliorated by high dose of noggin cICV (*P* = 0.771 for 500 ng/day and *P = 0.026 for 1000 ng/day). **L–N.** Western blot shows significantly increased Gfap levels after BCAS (**P < 0.001 for Sham vs. BCAS + ACSF), which is suppressed by noggin cICV (**P < 0.001 for BCAS + ACSF vs. BCAS + noggin 500 and 1000 ng/day) (L, M). Western blot also shows significantly decreased Olig2 levels after BCAS (*P = 0.030 for Sham vs. BCAS + ACSF), which is ameliorated by a high dose of noggin cICV (*P* = 0.698 for BCAS + ACSF vs. BCAS + noggin 500 ng/day; and *P = 0.042 for BCAS + ACSF vs. BCAS + noggin 1000 ng/day) (L, N). **O.** Representative images for MBP. *Bar* indicates 100 µm. **P.** Semiquantitative analysis of immunofluorescent images shows that BCAS significantly decreases Mbp (**P = 0.001), which is significantly ameliorated by high dose of noggin cICV (*P* = 0.169 for 500 ng/day and *P = 0.034 for 1000 ng/day). *Vertical bars* represent mean ± SEM. Abbreviations are as follows: B, BCAS; cICV, continuous intracerebroventricular infusion; IHC, immunohistochemistry; N500, noggin 500 ng/day; N1000, noggin 1000 ng/day; S, Sham operation; Tubj1, tubulin gamma 1, also known as gamma tubulin; WB, western blot.

Adult mice subjected to BCAS and noggin cICV (or ACSF only) treatment were analyzed by immunohistochemistry and western blot (Figure 6A,H). The inhibitory effect of noggin on BMP signaling was confirmed by a reduction of phosphorylated Smad immunoreactivity (data not shown).

Bmp4 was expressed in the Acta2/Pdgfrb double‐positive pericytes in capillaries of 28‐day‐BCAS mouse brains (Figure 6B). Western blot showed significantly increased expression of Bmp4 precursor (Figure 6C,D and *Supporting Information* Figure S4A) and Pdgfrb (Figure 6E,F and *Supporting Information* Figure S4B), but not Acta2 (Figure 6E,G and *Supporting Information* Figure S4C) after BCAS, compared with sham controls. The Bmp4 precursor 41 was detected by both C‐terminal (ab39973, Abcam, Cambridge, UK) and N‐terminal (MAB1049, Millipore, and sc‐393329, SantaCruz, Dallas, TX, USA) domain antibodies (data not shown). The expressions of western blot sample were normalized to tubulin gamma 1 (Tubg1) expression, which did not fluctuate under hypoperfusion.

The percent area of Gfap expression increased after BCAS, which was suppressed by noggin cICV (Figure 6I,J). The percent area of Olig2 expression was decreased by BCAS, which was ameliorated by noggin cICV at a dosage of 1000 ng/day (Figure 6I,K). Western blot also showed significantly increased Gfap expressions in the BCAS mice, which was suppressed by noggin cICV (both 500 ng/day and 1000 ng/day) (Figure 6L,M and *Supporting Information* Figure S4D). Olig2 expression was decreased by BCAS, which was ameliorated by a high dose of noggin cICV (1000 ng/day) (Figure 6L,N and *Supporting Information* Figure S4E). Each band of Gfap and Olig2 was normalized with non‐specific band stained with ponceau S staining (Figure 6L and *Supporting Information* Figure S4F). To assess the effect of BMP4 on mature oligodendrocytes, the percent area of Mbp expression was quantitated. The percent area of Mbp was decreased by BCAS, which was ameliorated by noggin cICV at a dosage of 1000 ng/day (Figure 6O,P).

## DISCUSSION

We previously reported that myelin loss evolved in parallel with shrunken oligodendrocytes in SVD compared with AD 20. Another study revealed that spongiosis, arteriolosclerosis, état criblé and myelin loss were more severe in the SVD cases than the AD cases 13, suggesting different etiologies underlie the white matter attenuation between SVD and AD. In this study, we clarified one of the causative factors underlying the different aspects of white matter changes: namely, BMP4 generated from pericytes. We analyzed expressions of six TGFB superfamily members in the brains of SVD, AD, and age‐matched controls, and found BMP4 was distinctly expressed in pericytes of the white matter. BMP4 expression was significantly upregulated in the SVD group, and was negatively correlated with myelin density. Consistent with these findings, long‐term OGD in cultured pericytes and *in vivo* chronic hypoperfusion induced BMP4 upregulation. Given that chronic hypoperfusion during late embryonic stages 36 and neonatal stages 12 increases BMP4 expression and impairs differentiation of OPCs, chronic hypoperfusion may also upregulate BMP4 in the elderly. Recent evidence suggests that Tgfb upregulation contributes to cerebrovascular dysfunction in mice 34, which ascertains CARASIL pathogenesis in humans through hereditary loss of *HtrA1* 18. Therefore, these results suggest upregulation of the TGFB superfamily members is a shared mechanism in the pathogenesis of ischemic cerebrovascular disorders, regardless of whether it is sporadic or hereditary.

Previous studies have shown that Bmps, including Bmp2, 4, 6 and 7, are upregulated in a variety of CNS injury and demyelinating disease animal models 14, 45. In this study, expression of all six TGFB superfamily members was also observed in macrophages and activated astrocytes within the vicinity of microinfarcts in the SVD group. However, in demyelinated but not infarcted areas, of the SVD group, BMP4 was almost exclusively expressed in pericytes.

The density of PDGFRB‐positive pericytes in the white matter correlated with myelin attenuation. In previous studies, PDGFRB immunostaining was used to assess microvasculature, showing a reduction of PDGFRB‐positive pericytes and a breakdown of the blood‐brain barrier in vascular cognitive impairment and some neurodegenerative diseases, including AD and ALS 46, 51, 55, 56, 59. However, a recent study showed increased expression of PDGFRB immunoreactive pericytes in cerebral microvessels in CADASIL (cerebral autosomal dominant arteriopathy with subcortical infarcts and leukoencephalopathy) compared with similar age controls 11. It has also been suggested that *Pdgfrb* mRNA is upregulated in pericytes in the infarcted area 37, and Pdgfrb‐positive pericytes migrate into the peri‐infarct area after stroke, contributing to angiogenesis and vascular remodeling 25. In the current study, Pdgfrb expression was also upregulated by chronic hypoperfusion in the BCAS mice. Given that the PDGFRB is important for proliferation and migration of pericytes 55, PDGFRB may be increased in pericytes at a certain point, as compensation for hypoperfusion.

Unlike PDGFRB, ACTA2 expression was not different between the three groups. In terms of tumor angiogenesis, Acta2 served as a mature pericyte marker whereas Pdgfrb served as a marker for progenitor perivascular cells with the ability to differentiate into pericytes and regulate vessel stability and vascular survival 50. Therefore, the differences in PDGFRB and ACTA2 expression may be the result of different stages of pericyte maturation; PDGFRB‐positive pericytes in the white matter are immature pericytes during angiogenesis following chronic hypoperfusion, and PDGFRB‐negative/ACTA2‐positive pericytes are mature pericytes in relatively intact white matter. Because BMP4 treatment facilitated pericyte proliferation and *Pdgfrb* mRNA expression, an increase in PDGFRB‐positive pericytes in the SVD group might be induced by BMP4 in response to chronic hypoperfusion. BMP4 also facilitated proliferation and tube formation of endothelial cells, which is another key factor for maintenance of vascular integrity. These results suggest that BMP4 induces inherent compensatory angiogenesis against chronic hypoperfusion. Despite the putative compensatory responses, COL4A1‐positive vessel density was not significantly different in SVD compared with AD or disease control patients in the cerebral cortex and white matter, suggesting that angiogenesis was not sufficient or compensatory enough to recruit blood vessels and mitigate cerebral hypoperfusion in SVD patients. Because time‐specific induction of BMP4 and PDGFRB is necessary for successful angiogenesis, long‐lasting hypoperfusion in the aged brain may block appropriate angiogenesis to restore regional blood flow 52.

With regard to myelination, BMP4 overexpression may not be preferable. BMP4 treatment has been shown to induce differentiation of oligodendroglial‐astroglial progenitor cells into astrocytes 17, 27. Our results also showed that BMP4 strongly suppressed maturation of primary OPCs and converted OPCs into astrocytes in differentiation media. Compared with 1.2‐ to 2‐fold changes in angiogenesis, the degree of inducing astrogliogenesis 49 and suppressing oligodendrogenesis was exponential. Furthermore, even when the OPCs were cultured in proliferation media which should maintain an OPC state, BMP4 treatment decreased *Pdgfra* mRNA expression in a dose‐dependent manner. This finding was inconsistent with other studies that demonstrated no effect 16, 57 or even a positive effect 43 of BMP4 on OPC proliferation. However, recent studies have suggested that the effects of BMP4 on stem cell proliferation are dose‐dependent; low dose BMP4 increases proliferation, but high dose BMP4 decreases proliferation of mesenchymal stem cells 53 or primordial germ cells 31. Considering that OPC proliferation is also affected by many factors, BMP4 may have dose‐dependent and opposite effects on OPC proliferation. Consistent with the results of cell culture, the number of OPCs in SVZ is decreased with higher BMP4 expression of SVD cases with severe astrogliosis. BCAS mice also showed astrogliosis and hypomyelination as shown previously 48. As noggin, a receptor antagonist of BMP4, suppressed astrogliogenesis and induced oligodendrogenesis *in vitro*, both high and low doses of noggin cICV decreased the number of astrocytes and high dose of noggin cICV increased the number of oligodendrocytes.

The close relationship between BMP4 and myelin attenuation is also supported by recent studies 3, 15, 26, 43. These data strongly suggest that BMPs are involved in oligovascular pathologies in the brain. Considering a potential anatomical and functional interaction between pericytes and OPCs in the capillary of cerebral white matter 30, the pericyte‐induced BMP4 might directly affect OPC lineage. Because many factors should be expressed in a coordinated manner after cerebral hypoperfusion, time‐specific control of BMP expression should be further investigated to protect oligovascular units in ischemic demyelinating diseases.

The main limitation of this study was the lack of clinical and neuropsychometric information on the postmortem brains used. This meant that we could not directly relate our pathological findings to antemortem hypoperfusive and cognitive status. In addition, the lack of comparison of the SVD cases with controls without neurodegenerative pathologies was another limitation considering that PDGFRB positive pericytes may also degenerate in some neurodegenerative diseases 46, 55, 56, 59. The third limitation is that a histopathological study using postmortem human brains does not always uncover the entire cell process that occurs in the ageing human brain. Although the process was at least partially modeled by both cultured pericytes under OGD and mice subjected to BCAS, future studies should focus on examining dynamic changes of pericyte disruption and BMP4 values in the disease course of SVD using neuroimaging and biomarkers.

**Figure 7:**
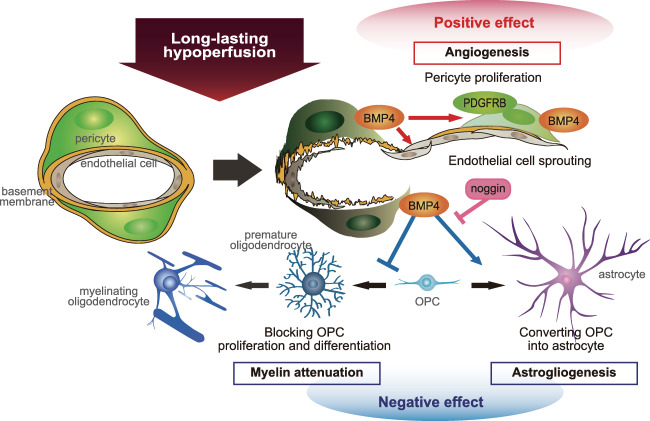
Putative scheme for BMP4 effect on multiple cell lineages after chronic ischemia. BMP4 generated from pericytes after hypoperfusion may promote compensatory angiogenesis by significantly increasing production of endothelial cells and pericytes. However, BMP4 expression may also induce astrogliogenesis at the expense of OPC proliferation and maturation, thereby aggravating white matter damage. Noggin has the potential to suppress BMP4‐induced astrogliogenesis and rescue oligodendrogenesis.

In summary, our study suggests that during chronic hypoperfusion akin to that in SVD, BMP4 is generated in pericytes predominantly in the white matter, providing putative compensatory angiogenesis. However, BMP4 also aggravates white matter damage by inducing astrogliogenesis at the expense of OPC proliferation and maturation. Based on our observations, we propose a putative scheme how these cellular processes could occur (Figure 7). This may explain why white matter is particularly vulnerable to chronic hypoperfusion.

## AUTHOR CONTRIBUTIONS

M.T.U. study design, acquisition and analysis of data, and drafting the manuscript and figures; M.I. study conception and design, handling funding and supervising portions of the study; T.M. design of the study, and supervising and making critical revision of the manuscript for important intellectual content; T.N. data acquisition and analysis, and supervising and making critical revision of the manuscript for important intellectual content; S.K. data acquisition and analysis; K.U. and A.K. supervising and making critical revision of the manuscript for important intellectual content.; R.N.K. and R.T. handling funding, supervising and making critical revision of the manuscript for important intellectual content.

## Supporting information

Additional Supporting Information may be found in the online version of this article at the publisher's web‐site:

**Figure S1.** Expression of TGFB1, BMP2, BMP6, BMP7, and BMP9 in white matter. Representative images of TGFB1, BMP2, BMP6, BMP7, and BMP9 staining in the WM of control, AD, and SVD, respectively. Insets show TGFB1, BMP2, BMP6, BMP7, or BMP9‐positive activated astrocytes with large cell bodies (right panels), TGFB1 or BMP7‐positive pericytes (left panels in TGFB1 and BMP7), and BMP2‐positive endothelial cells (left panels in BMP2), respectively. *Bars* indicate 100 µm and 10 µm (insets).
**Figure S2.** Oligodendrocyte precursor cells in the subventricular zone. Representative images for PDGFRA‐positive cells surrounding arterioles, venules and capillaries in the subventricular zone. *Bars* indicate 25 µm.
**Figure S3.** Effects of BMP4 on oligodendrocyte maturation in the OPC differentiation assay. (A) Time‐lapse images of primary OPCs in differentiation media with or without BMP4. Insets show enlarged images. *Bars* indicate 100 μm and 50 μm (insets). (B) Representative triple‐immunofluorescent images for Mbp and Gfap, and their merged images with DAPI for nuclear staining. Insets show enlarged images. *Bars* indicate 100 µm and 15 µm (insets). (C, D) Relative mRNA levels of *Mbp* and *Gfap* in the differentiation assay. *Mbp* mRNA levels decrease in differentiation media containing BMP4 (**P < 0.001 for 1 and 10 ng/ml) in a dose‐dependent manner; the effect of BMP4 is reversed by noggin (**P < 0.001). Treatment with noggin alone increases *Mbp* (**P < 0.001) (C). BMP4 significantly increases *Gfap* mRNA expression (**P < 0.001 for 1 and 10 ng/ml) in a dose‐dependent manner; the effect of BMP4 is canceled out by noggin (**P < 0.001) (D). *Vertical bars* represent mean ± SD. Abbreviation is as follow: Cont, control; Gapdh, glyceraldehyde‐3‐phosphate dehydrogenase.
**Figure S4.** Original gel pictures for western blots. The figure shows uncropped western blots displayed in Fig. 6C. (A) Bmp4 precursor and pro‐Bmp4 expressions are increased in BCAS mice compared with sham controls. Pdgfrb (B), but not Acta2 (C), expression is increased in BCAS mice compared with sham controls. Each band of Bmp4, Pdgfrb, and Acta2 is normalized to Tubg1. (D) Gfap expressions are increased in BCAS mice compared with sham controls, which are suppressed by noggin cICV (500 ng/day and 1000 ng/day). (E) Olig2 expressions are decreased in BCAS mice compared with sham controls, which are ameliorated by a high dose of noggin cICV (1000 ng/day). (F) Each band of Gfap and Olig2 is normalized to the band at 45 kDa stained with ponceau S. Asterisks show unknown bands. Abbreviations are as follows: B, BCAS; N500, Noggin 500 ng/day; N1000, Noggin 1000 ng/day; S, Sham operation.
**Table S1.** Primary antibodies used for immunohistochemistry of human brain tissue.
**Table S2.** Primers used for RT‐PCR.
**Table S3.** Primary antibodies used for immunocytochemistry.
**Table S4.** Primary antibodies used for immunohistochemistry of mouse brain tissue.
**Table S5.** Primary antibodies used for western blot.

Supporting Movie S1

Supporting Movie S2
