# Supplementary material for: Pericyte‐derived bone morphogenetic protein 4 underlies white matter damage after chronic hypoperfusion
Source: Brain Pathol. 2017 May 31;28(4):521–35. doi: 10.1111/bpa.12523 (PMC6099372; doi:10.1111/bpa.12523)
Supplement: Supplementary file 1 — Figure S1. Expression of TGFB1, BMP2, BMP6, BMP7, and BMP9 in white matter. Representative images of TGFB1, BMP2, BMP6, BMP7, and BMP9 staining in the WM of control, AD, and SVD, respectively. Insets show TGFB1, BMP2, BMP6, BMP7, or BMP9‐positive activated astrocytes with large cell bodies (right panels), TGFB1 or BMP7‐positive pericytes (left panels in TGFB1 and BMP7), and BMP2‐positive endothelial cells (left panels in BMP2), respectively. Bars indicate 100 µm and 10 µm (insets). Figure S2. Oligodendrocyte precursor cells in the subventricular zone. Representative images for PDGFRA‐positive cells surrounding arterioles, venules and capillaries in the subventricular zone. Bars indicate 25 µm. Figure S3. Effects of BMP4 on oligodendrocyte maturation in the OPC differentiation assay. (A) Time‐lapse images of primary OPCs in differentiation media with or without BMP4. Insets show enlarged images. Bars indicate 100 μm and 50 μm (insets). (B) Representative triple‐immunofluorescent images for Mbp and Gfap, and their merged images with DAPI for nuclear staining. Insets show enlarged images. Bars indicate 100 µm and 15 µm (insets). (C, D) Relative mRNA levels of Mbp and Gfap in the differentiation assay. Mbp mRNA levels decrease in differentiation media containing BMP4 (**P < 0.001 for 1 and 10 ng/ml) in a dose‐dependent manner; the effect of BMP4 is reversed by noggin (**P < 0.001). Treatment with noggin alone increases Mbp (**P < 0.001) (C). BMP4 significantly increases Gfap mRNA expression (**P < 0.001 for 1 and 10 ng/ml) in a dose‐dependent manner; the effect of BMP4 is canceled out by noggin (**P < 0.001) (D). Vertical bars represent mean ± SD. Abbreviation is as follow: Cont, control; Gapdh, glyceraldehyde‐3‐phosphate dehydrogenase. Figure S4. Original gel pictures for western blots. The figure shows uncropped western blots displayed in Fig. 6C. (A) Bmp4 precursor and pro‐Bmp4 expressions are increased in BCAS mice compared with sham controls. Pdgfrb (B), but not Ac [file BPA-28-521-s002.docx]

**Supporting Information**

Uemura, et al.: **Pericyte-derived Bone Morphogenetic Protein 4 Underlies White Matter Damage after Chronic Hypoperfusion**

Corresponding author: Masafumi Ihara, MD, PhD, FACP

Department of Neurology, National Cerebral and Cardiovascular Center Hospital, Osaka, Japan

5-7-1 Fujishiro-dai, Suita, Osaka 565-8565, Japan

FAX: +81-6-6835-5137

TEL: +81-6-6833-5012

E-mail address: [ihara@ncvc.go.jp](mailto:ihara@ncvc.go.jp)

**Table S1. Primary antibodies used for immunohistochemistry of human brain tissue tissue**

| **Antigen** | **Host** | **Dilution** | **Product Code** | **Company** |
| --- | --- | --- | --- | --- |
| ACTA2 | Rabbit | 1:100 | ab5694 | Abcam, Cambridge, UK |
| BMP2 | Rabbit | 1:200 | ab82511 | Abcam |
| BMP4 | Mouse | 1:100 | MAB1049 | Millipore, Billerica, MA, USA |
| BMP6 | Mouse | 2 µg/ml | MAB1048 | Millipore |
| BMP7 | Rabbit | 4 µg/ml | ab56023 | Abcam |
| BMP9 | Rabbit | 1:50 | ab35088 | Abcam |
| COL4A1 | Rabbit | 1:200 | ab6586 | Abcam |
| MBP | Mouse | 1:200 | MA1-10837 | Thermo Fisher Scientific, Waltham, MA, USA |
| PDGFRA | Rabbit | 1:150 | 5241 | Cell Signaling, Danvers, MA, USA |
| PDGFRB | Goat | 1:500 | AF385 | R&D Systems, Minneapolis, MN, USA |
| TGFB1 | Mouse | 1:50 | sc-146 | Santa Cruz, Dallas, TX, USA |

**Table S2. Primers used for RT-PCR**

| **Gene** | **Species** | **Sequence (5' -> 3')** | |
| --- | --- | --- | --- |
| *Acta2* | Mouse | Fw | GGACGTACAACTGGTATTGTGC |
|  |  | Rv | TCGGCAGTAGTCACGAAGGA |
| *Bmp4* | Mouse | Fw | ATTCCTGGTAACCGAATGCTG |
|  |  | Rv | CCGGTCTCAGGTATCAAACTAGC |
| *Gapdh* | Mouse/Rat | Fw | TGACGTGCCGCCTGGAGAAA |
|  |  | Rv | AGTGTAGCCCAAGATGCCCTTCAG |
| *Gfap* | Rat | Fw | AGAAAACCGCATCACCATTC |
|  |  | Rv | GCACACCTCACATCACATCC |
| *Hprt* | Mouse | Fw | CTGGTGAAAAGGACCTCTCGAA |
|  |  | Rv | CTGAAGTACTCATTATAGTCAAGGGCAT |
| *Mbp* | Rat | Fw | ACACACAAGAACTACCCACTACGG |
|  |  | Rv | AGCTAAATCTGCTGAGGGACAG |
| *Pdgfra* | Rat | Fw | CTAATTCACATTCGGAAGGTTG |
|  |  | Rv | GGACGATGGGCGACTAGAC |
| *Pdgfrb* | Mouse | Fw | ACAATTCCGTGCCGAGTGACAG |
|  |  | Rv | AAAAGTACCAGTGAAACCTCGCTG |

**Table S3. Primary antibodies used for immunocytochemistry**

| **Antigen** | **Host** | **Dilution** | **Product Code** | **Company** |
| --- | --- | --- | --- | --- |
| Gfap | Rabbit | 1:500 | Z0334 | DAKO, Glostrup, Denmark |
| Mbp | Mouse | 1:200 | MA1-10837 | Thermo Fisher Scientific |
| Pdgfra | Goat | 1:200 | AF1062 | R&D Systems |
| PECAM1 | Mouse | 1:1000 | 3528 | Cell Signaling |

**Table S4. Primary antibodies used for immunohistochemistry of mouse brain tissue**

| **Antigen** | **Host** | **Dilution** | **Product Code** | **Company** |
| --- | --- | --- | --- | --- |
| Acta2 | Rabbit | 1:100 | ab5694 | Abcam |
| Bmp4 | Mouse | 1:100 | MBA1049 | Millipore |
| Gfap | Rat | 1:200 | 13-0300 | Thermo Fisher Scientific |
| Mbp | Rabbit | 1:200 | PD004 | MBL, Nagoya, Japan |
| Olig2 | Rabbit | 1:500 | AB9610 | Millipore |
| Pdgfrb | Goat | 1:1000 | AF1042 | R&D Systems |

**Table S5. Primary antibodies used for western blot**

| **Antigen** | **Host** | **Dilution** | **Product Code** | **Company** |
| --- | --- | --- | --- | --- |
| Acta2 | Rabbit | 1:1000 | ab5694 | Abcam |
| *Bmp4 | Rabbit | 1:1000 | ab39973 | Abcam |
| Bmp4 | mouse | 1:1000 | MAB1049 | Millipore |
| Bmp4 | mouse | 1:1000 | sc-393329 | Santa Cruz, Dallas, TX, USA |
| Gfap | Rabbit | 1:1000 | Z0334 | DAKO |
| Olig2 | Rabbit | 1:2000 | AB9610 | Millipore |
| Pdgfrb | Goat | 1:1000 | AF1042 | R&D Systems |
| Tubg1 | Mouse | 1:10000 | T-6557 | Sigma, Saint Louis, MO, USA |

* used in Fig. 6C and Supplementary Fig. 2A.


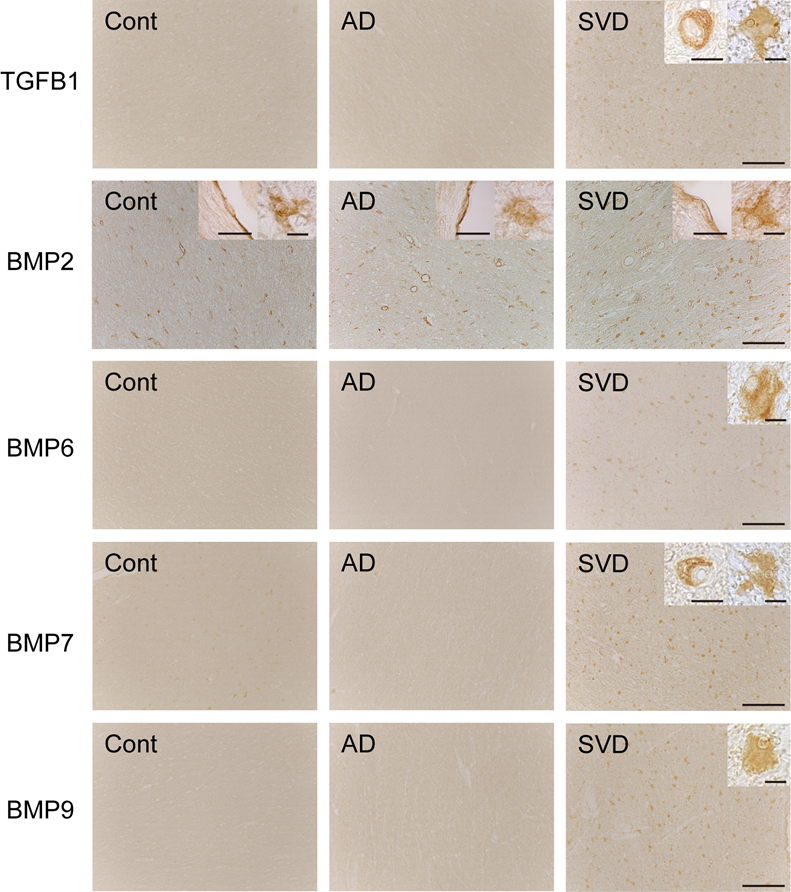


**Figure S1. Expression of TGFB1, BMP2, BMP6, BMP7, and BMP9 in white matter.** Representative images of TGFB1, BMP2, BMP6, BMP7, and BMP9 staining in the WM of control, AD, and SVD, respectively. Insets show TGFB1, BMP2, BMP6, BMP7, or BMP9-positive activated astrocytes with large cell bodies (right panels), TGFB1 or BMP7-positive pericytes (left panels in TGFB1 and BMP7), and BMP2-positive endothelial cells (left panels in BMP2), respectively. *Bars* indicate 100 µm and 10 µm (insets).

**
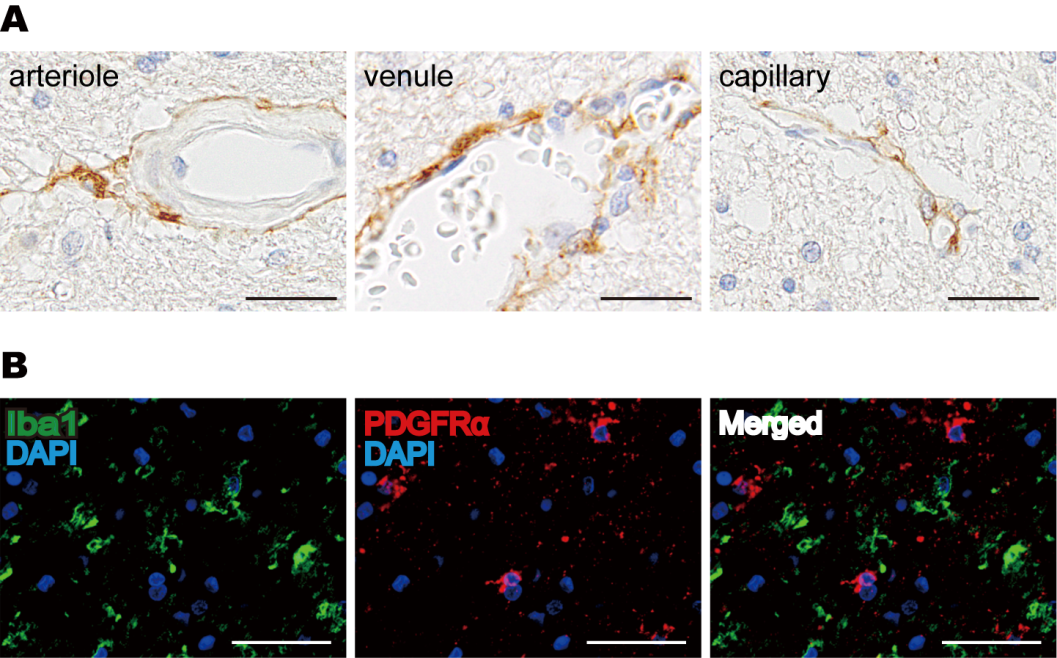
**

**Figure S2. Oligodendrocyte precursor cells in the subventricular zone.** Representative images for PDGFRA-positive cells surrounding arterioles, venules and capillaries in the subventricular zone. *Bars* indicate 25 µm.


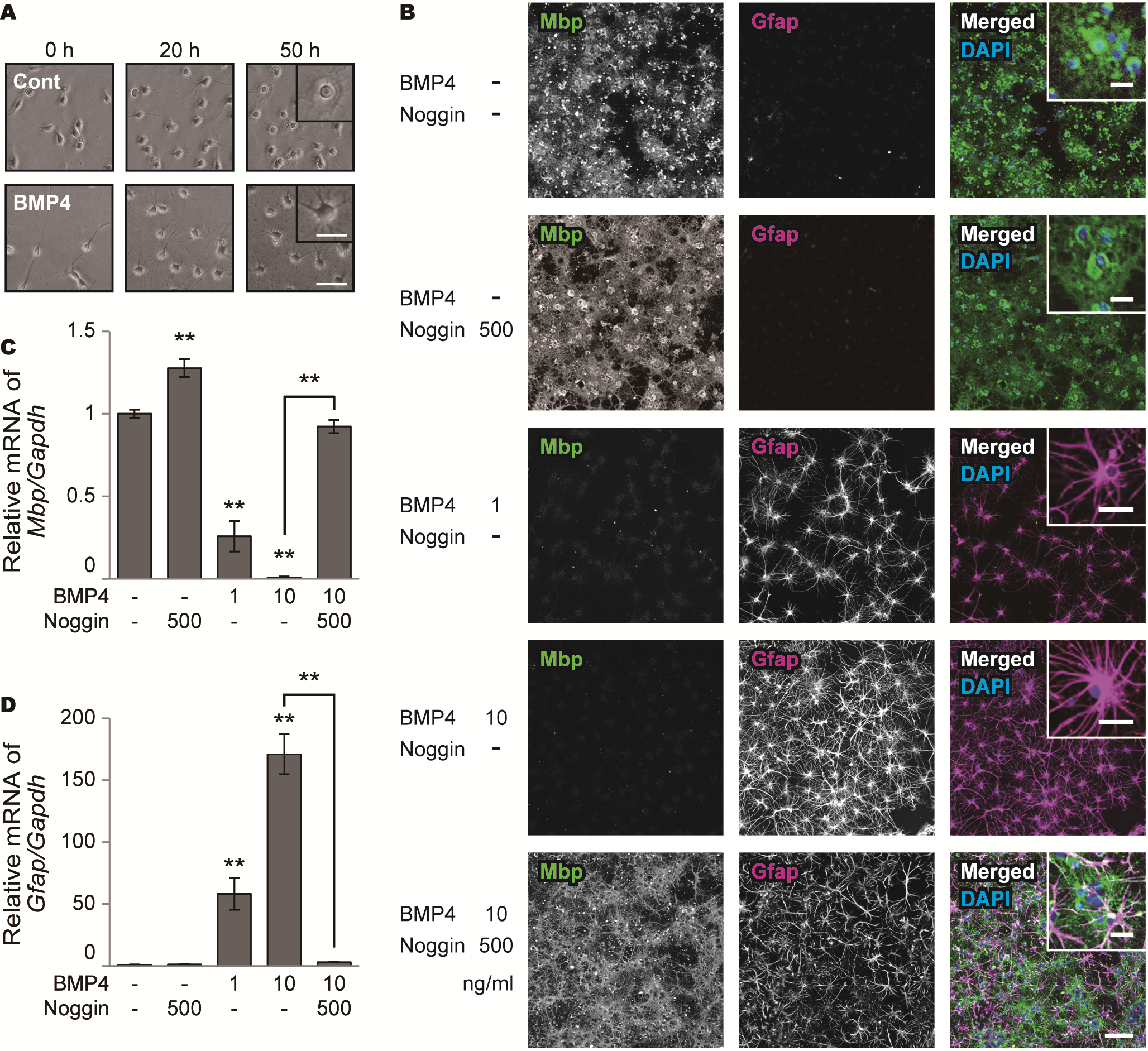


**Figure S3. Effects of BMP4 on oligodendrocyte maturation in the OPC differentiation assay.** (A) Time-lapse images of primary OPCs in differentiation media with or without BMP4. Insets show enlarged images. *Bars* indicate 100 μm and 50 μm (insets). (B) Representative triple-immunofluorescent images for Mbp and Gfap, and their merged images with DAPI for nuclear staining. Insets show enlarged images. *Bars* indicate 100 µm and 15 µm (insets). (C, D) Relative mRNA levels of *Mbp* and *Gfap* in the differentiation assay. *Mbp* mRNA levels decrease in differentiation media containing BMP4 (***P* < 0.001 for 1 and 10 ng/ml) in a dose-dependent manner; the effect of BMP4 is reversed by noggin (***P* < 0.001). Treatment with noggin alone increases *Mbp* (***P* < 0.001) (C). BMP4 significantly increases *Gfap* mRNA expression (***P* < 0.001 for 1 and 10 ng/ml) in a dose-dependent manner; the effect of BMP4 is canceled out by noggin (***P* < 0.001) (D). *Vertical bars* represent mean ± SD. Abbreviation is as follow: Cont, control; Gapdh, glyceraldehyde-3-phosphate dehydrogenase.


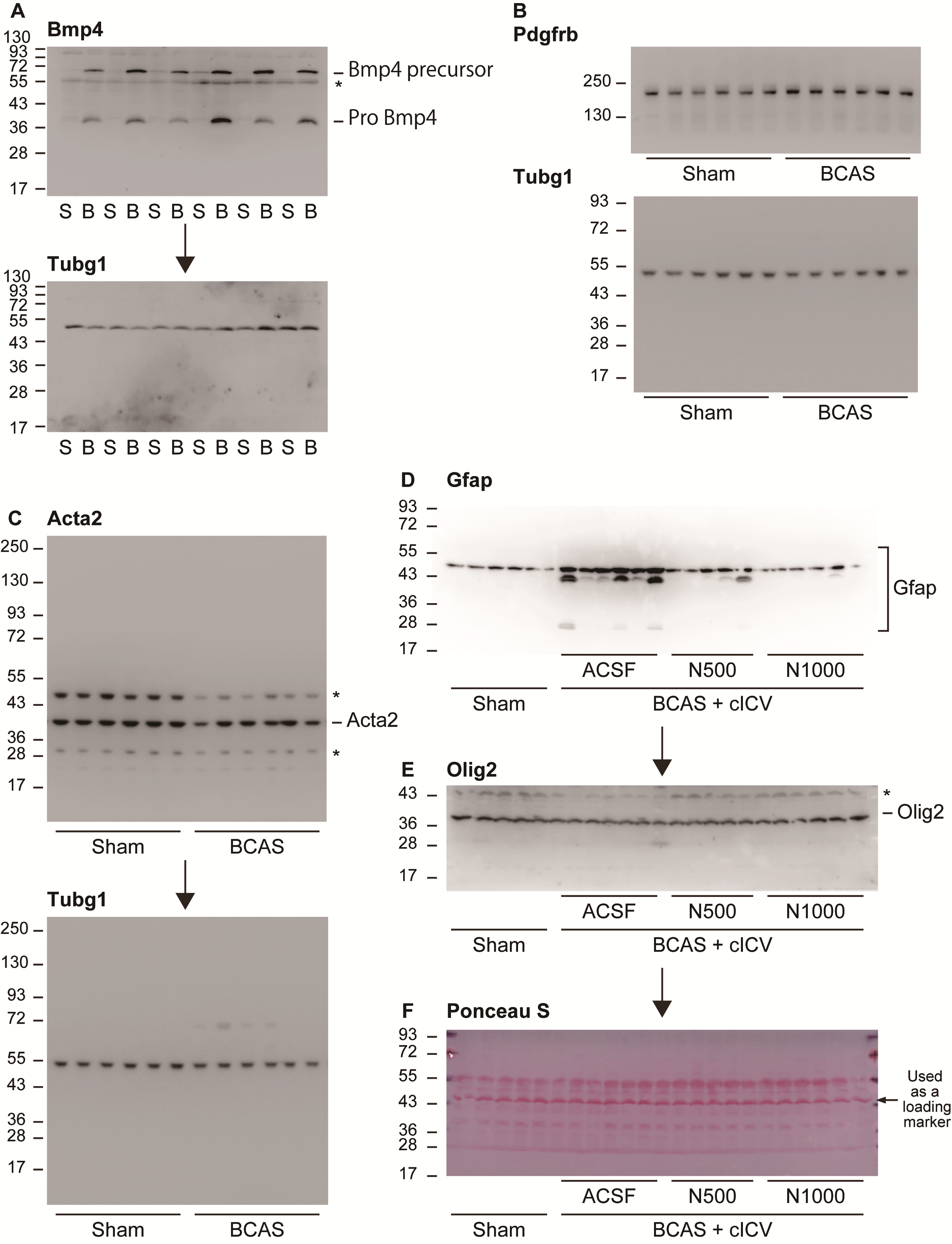


**Figure S4. Original gel pictures for western blots.** The figure shows uncropped western blots displayed in Fig. 6C. **(**A) Bmp4 precursor and pro-Bmp4 expressions are increased in BCAS mice compared with sham controls. Pdgfrb (B), but not Acta2 (C), expression is increased in BCAS mice compared with sham controls. Each band of Bmp4, Pdgfrb, and Acta2 is normalized to Tubg1. (D) Gfap expressions are increased in BCAS mice compared with sham controls, which are suppressed by noggin cICV (500 ng/day and 1000 ng/day). (E) Olig2 expressions are decreased in BCAS mice compared with sham controls, which are ameliorated by a high dose of noggin cICV (1000 ng/day). (F) Each band of Gfap and Olig2 is normalized to the band at 45 kDa stained with ponceau S. Asterisks show unknown bands. Abbreviations are as follows: B, BCAS; N500, Noggin 500 ng/day; N1000, Noggin 1000 ng/day; S, Sham operation.
